# Impact of the COVID-19 pandemic on prehospital and in-hospital treatment and outcomes of patients after out-of-hospital cardiac arrest: a Japanese multicenter cohort study

**DOI:** 10.1186/s12873-024-00929-8

**Published:** 2024-01-08

**Authors:** Chie Tanaka, Takashi Tagami, Junya Kaneko, Nobuya Kitamura, Hideo Yasunaga, Shotaro Aso, Munekazu Takeda, Masamune Kuno

**Affiliations:** 1 Department of Emergency and Critical Care Medicine, Nippon Medical School Tama Nagayama Hospital, Tama-shi, Tokyo 2068512 Japan; 2 Department of Emergency and Critical Care Medicine, Nippon Medical School Musashikosugi Hospital, 1-396 Kosugimachi, Nakahara-ku, Kawasaki, Kanagawa 211-8533 Japan; 3 Department of Clinical Epidemiology and Health Economics, School of Public Health, The University of Tokyo, Bunkyo, Tokyo 1138654 Japan; 4 Department of Emergency and Critical Care Medicine, Kimitsu Chuo Hospital, Kimitsu, Chiba 2928535 Japan; 5 Department of Real-world Evidence, Graduate School of Medicine, The University of Tokyo, Tokyo, 1138654 Japan; 6 Department of Critical Care and Emergency Medicine, Tokyo Women’s Medical University, Tokyo, 1628666 Japan

**Keywords:** Out-of-hospital cardiac arrest, COVID-19, Chain of survival, Mortality

## Abstract

**Background:**

In the chain of survival for Out-of-hospital cardiac arrest (OHCA), each component of care contributes to improve the prognosis of the patient with OHCA. The SARS-CoV-2 (COVID-19) pandemic potentially affected each part of care in the chain of survival. The aim of this study was to compare prehospital care, in-hospital treatment, and outcomes among OHCA patients before and after the COVID-19 pandemic.

**Methods:**

We analyzed data from a multicenter prospective study in Kanto area, Japan, named SOS-KANTO 2017. We enrolled patients who registered during the pre-pandemic period (September 2019 to December 2019) and the post-pandemic period (June 2020 to March 2021). The main outcome measures were 30-day mortality and the proportion of favorable outcomes at 1 month, and secondary outcome measures were changes in prehospital and in-hospital treatments between the pre- and post-pandemic periods.

**Results:**

There were 2015 patients in the pre-pandemic group, and 5023 in the post-pandemic group. The proportion of advanced airway management by emergency medical service (EMS) increased (*p* < 0.01), and EMS call-to-hospital time was prolonged (*p* < 0.01) in the post- versus pre-pandemic group. There were no differences between the groups in defibrillation, extracorporeal membrane oxygenation, or temperature control therapy (*p* = 0.43, *p* = 0.14, and *p* = 0.16, respectively). Survival rate at 1 month and favorable outcome rate at 1 month were lower (*p* = 0.01 and *p* < 0.01, respectively) in the post- versus pre-pandemic group.

**Conclusion:**

Survival rate and favorable outcome rate 1 month after return of spontaneous circulation of OHCA worsened, EMS response time was prolonged, and advanced airway management by EMS increased in the post- versus pre-pandemic group; however, most prehospital and in-hospital management did not change between pre- and post-COVID-19 pandemic.

**Supplementary Information:**

The online version contains supplementary material available at 10.1186/s12873-024-00929-8.

## Background

Out-of-hospital cardiac arrest (OHCA) is a leading cause of death and a major public health problem. In the chain of survival for OHCA, each component of care— recognition, calling the emergency medical service (EMS), starting cardiopulmonary resuscitation (CPR) by a bystander, advanced life support and transportation to the hospital by EMS, and post-cardiac arrest advanced care by hospital professionals—contributes to improve the prognosis of the patient with OHCA [1].

On 11 March 2020, the World Health Organization declared the SARS-CoV-2 (COVID-19) pandemic [2], and it drastically changed not only the health and lives of individual patients, but also public health, society, the economy, and medical services. The COVID-19 pandemic potentially affected each part of care in the chain of survival, and many studies from regions and countries worldwide have already shown the effects of COVID-19 on OHCA care [3–5]. Most of these studies have focused on patient demographics, data on bystander CPR or automated external defibrillator (AED) use by citizens, prehospital care by EMS providers, and outcomes. Few studies have included both prehospital and in-hospital information on OHCA patients [6]; however, to evaluate the actual effect of the COVID-19 pandemic on OHCA care, we must consider not only prehospital situations but also in-hospital conditions.

For the current analysis, we aimed to compare prehospital care, in-hospital treatment, and outcomes among OHCA patients before and after the COVID-19 pandemic, using data from the multicenter prospective cohort study in Kanto area, Japan.

## Methods

### Study design

The current study was a post hoc analysis of SOS-KANTO 2017, a prospective cohort study carried out in the Kanto region in Japan and supported by the Kanto Regional Group of the Japanese Association for Acute Medicine. This study collected prehospital records of cardiac arrest (CA) patients transported to participating hospitals by trained EMS providers and admitted. This database contained information about patients’ vital signs on scene, patients’ background, witnesses, bystander CPR, initial rhythm documented by EMS, treatments by EMS, the cause of OHCA, vital signs on arrival at hospital, treatments in the hospital, onset time, hospital arrival time, neurological outcomes, and death [7–9].

### Definitions

The definition of CA was an absence of pulse and normal breathing [10].

The study period of SOS-KANTO 2017 was from September 2019 to March 2021. To define before and after COVID-19 pandemic, we classified the study period into three in accordance with the epidemic trends of COVID-19 in Japan: pre-COVID-19 pandemic (September 2019 to December 2019; hereafter “pre-pandemic”), the transitional time from the beginning to the spread of COVID-19 in Japan (January 2020 to May 2020), and post-COVID-19 pandemic (June 2020 to March 2021; hereafter “post-pandemic”) [11].

This study included information on neurological outcomes at discharge from hospital using the cerebral performance category (CPC) score, as follows: 1, good performance; 2, moderate disability; 3, severe disability; 4, vegetative state; and 5, death [10]. We grouped CPC 1 and 2 to denote a favorable outcome, and CPC 3, 4, and 5 to denote a poor outcome, in accordance with previous studies [10].

### Patient selection

Of all OHCA patients registered in SOS-KANTO 2017 conducted from September 2019 to March 2021, we enrolled patients who registered during the pre-pandemic period (September 2019 to December 2020) and post-pandemic period (June 2020 to March 2021).

### Outcome measures

Main outcome measures were 30-day mortality and the proportion of favorable outcomes (CPC 1 and 2) at 1 month. Secondary outcome measures were the rate of prehospital return of spontaneous circulation (ROSC), prehospital treatments, and in-hospital treatments.

### Statistical analysis

We compared patients’ backgrounds and covariates between the pre- and post-pandemic groups. Results were expressed as the median (interquartile range [IQR]) for non-normally distributed data. The analysis of continuous variables was conducted using Student’s *t*-test or Mann-Whitney U test, and categorical variables were compared with the chi-squared test or Fisher’s test, as appropriate. Next, we compared prehospital treatments, in-hospital treatments, death, and neurological outcomes using the same methods.

Further, to decrease the bias caused by incomplete data, we conducted multiple imputation: each missing value was replaced with a set of five substitute plausible values, and one model was created by statistical inference with the results of the five imputed data sets using a Markov chain Monte Carlo algorithm known as chained equations imputation [12, 13]. Finally, we performed multivariable regression analysis adjusting for the variables that were independently associated with death in OHCA patients, in accordance with the previous studies, and for within-hospital clustering using the generalized estimation equation. The variables were as follows: pre- or post-pandemic period, age, sex, witnessed status, bystander CPR, initial rhythm, location of arrest, and EMS time from to call to hospital [4, 5, 14]. The statistical significance threshold was *p* < 0.05. All data were analyzed using SPSS software (version 28; IBM Corp., Armork, NY, USA).

## Results

**Fig. 1:**
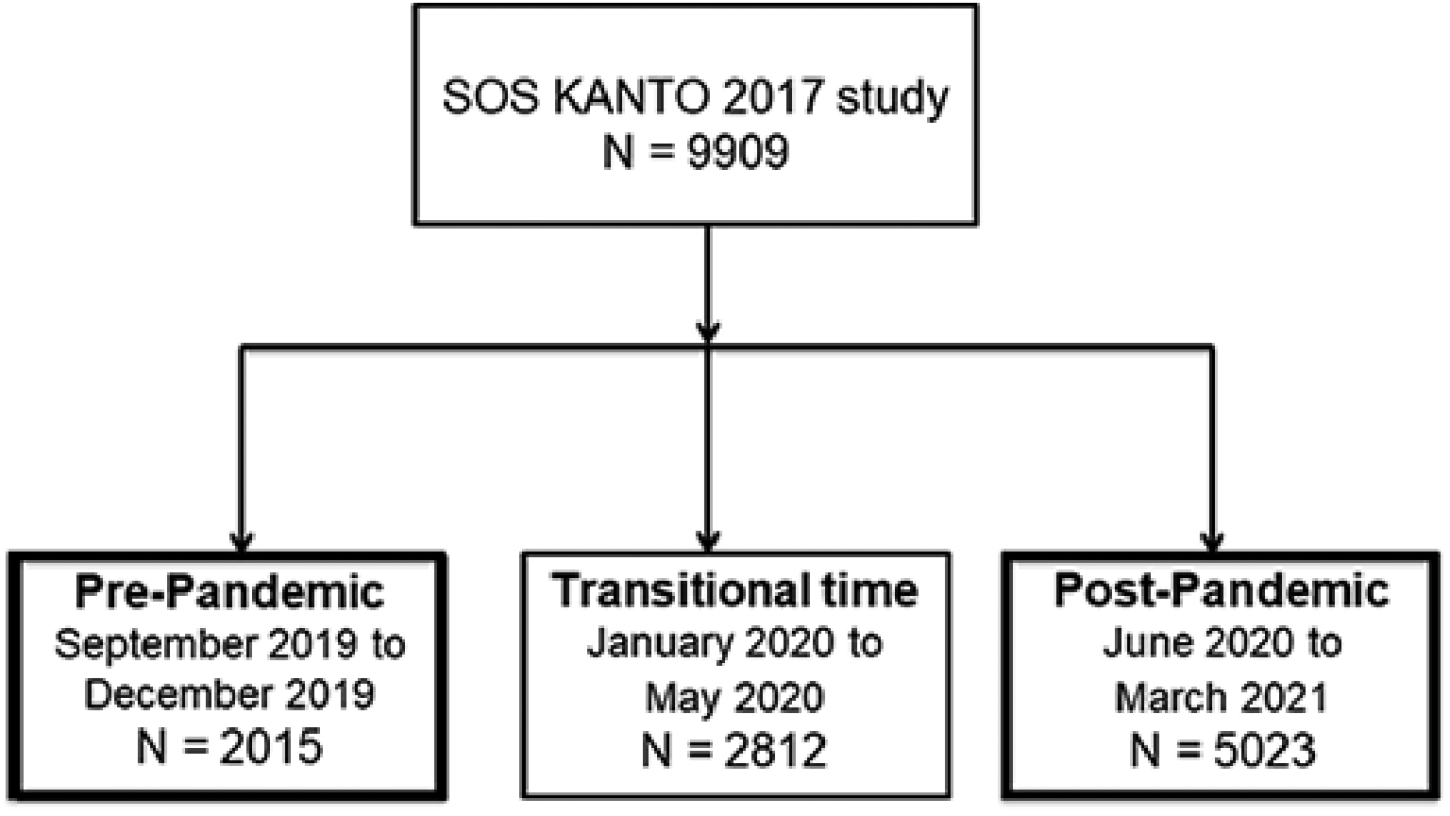
Patient selection

Among 9909 patients in the SOS-KANTO 2017 studies, there were 2015 patients in the pre-pandemic group, and 5023 in the post-pandemic group (Fig. 1).

### Baseline characteristics

Table 1 shows the patients’ characteristics. There were no significant differences in age (76 years [IQR: 62–84] vs. 72 years [IQR: 62–84]; *p* = 0.09) or sex (male) (61.6.% vs. 61.6%; *p* = 0.85) between groups. Most of the other factors were similar between the two groups; however, the proportions of patients with mild disability and the proportions of patients from healthcare facilities were slightly increased in the post-pandemic group versus pre-pandemic group. There was no statistical difference between the two groups (61.6% vs. 59.9%; *p* = 0.18) in cardiac cause of CA.

**Table 1:** Clinical characteristics of patients before and after the COVID-19 pandemic

Variable	Pre-pandemic (*n* = 2015)		Post-pandemic (*n* = 5023)		p-value
Age, years	72	(62–84)	76	(62–84)	0.09
Male	1236/2014	(61.4)	3095/5023	(61.6)	0.85
Body mass index, kg/m^2^	21.8	(19.1–25.0)	21.8	(18.8–25.4)	0.61
**Medical history**					
Hypertension	154/1945	(7.9)	332/4885	(6.8)	0.10
Diabetes	104/1941	(5.4)	187/4883	(3.8)	0.01
Ischemic heart disease	65/1938	(3.4)	100/4883	(2.0)	< 0.01
Cerebrovascular disease	54/1939	(2.8)	111/4886	(2.3)	0.21
Heart failure	53/1932	(2.7)	92/4781	(1.9)	0.03
Renal disease	49/1941	(2.5)	86/4889	(1.8)	0.04
Mental disease	35/1944	(1.8)	65/4890	(1.3)	0.14
Lung disease	30/1940	(1.5)	72/4887	(1.5)	0.82
Hemodialysis	24/1948	(1.2)	33/4899	(0.7)	0.02
Malignant disease	17/1940	(0.9)	46/4891	(0.9)	0.80
Liver disease	13/1940	(0.7)	27/4890	(0.6)	0.57
Digestive disease	12/1938	(0.6)	18/4883	(0.4)	0.16
**Baseline ADL**					< 0.01
Good ability	831/1406	(59.1)	1958/3669	(53.4)	
Mild disability	384/1406	(27.3)	1162/3669	(31.7)	
Severe disability	127/1406	(9.0)	322/3669	(8.8)	
Vegetative state	64/1406	(4.6)	227/3669	(6.2)	
Location					< 0.01
Home	1301/1996	(65.2)	3164/4673	(67.7)	
Public place	416/1996	(20.8)	807/4673	(17.3)	
Health care facilities	173/1996	(8.7)	470/4673	(10.0)	
Medical institutions	26/1996	(1.3)	49/4673	(1.0)	
Others	80/1996	(4.0)	183/4673	(3.9)	
**Cause of cardiac arrest**					
Cardiac etiology	1206/2015	(59.9)	3094/5023	(61.6)	0.18
Acute coronary syndrome	161/1197	(13.5)	290/3047	(9.3)	< 0.01
Noncardiac etiology	809/2015	(40.1)	1929/5023	(38.4)	0.16
Stroke	75/797	(9.5)	144/1895	(7.6)	
Respiratory disease	85/797	(10.7)	244/1895	(12.9)	
Malignancy	36/797	(4.5)	137/1895	(7.2)	
Exogenous cause	398/797	(49.9)	886/1895	(46.8)	< 0.01
Traffic accident	52/399	(13.0)	94/892	(10.5)	
Fall	62/399	(15.5)	171/892	(19.2)	
Hanging	70/399	(17.5)	203/892	(22.8)	
Drowning	44/399	(11.0)	103/892	(11.5)	
Asphyxia	124/399	(31.1)	225/892	(25.2)	
Addiction	14/399	(3.5)	10/892	(1.1)	
SIDS	11/2105	(0.5)	7/5023	(0.1)	< 0.01

Data are shown as the number of positive observations/total number of observations (%) or as median (interquartile range). For each variable, the number of missing observations can be obtained as the difference between the total number of patients in each group and the total number of observations

ADL, activities of daily living; SIDS, sudden infant death syndrome

### Prehospital information and treatments

Prehospital information is shown in Table 2. There were no statistical differences between groups in the witnessed by layperson (54.5% vs. 53.2%; *p* = 0.51), bystander CPR (45.3% vs. 43.7%; *p* = 0.23), and defibrillation using an AED performed by a bystander (2.6% vs. 2.2%; *p* = 0.36). The initial rhythm observed by paramedics did not differ between the groups (*p* = 0.13). For treatments performed by EMS, the proportion of advanced airway management was higher (*p* < 0.01) and EMS call-to-hospital time longer (35.0 min [IQR: 29.0–43.0] vs. 33.0 min [IQR: 27.0–40.0]; *p* < 0.01) in the post-pandemic group compared to the pre-pandemic group. The frequency of doctor ambulance/helicopter decreased significantly (9.2% vs. 6.4%; *p* < 0.01) in the post- versus pre-pandemic group.

**Table 2:** Prehospital demographics and treatments of patients before and after the COVID-19 pandemic

Variable	Pre-pandemic (n = 2015)		Post-pandemic (n = 5023)		p-value
Witnessed by layperson	802/1947	(53.2)	1866/4706	(54.5)	0.51
Witnessed by EMS	110/1947	(5.6)	274/4706	(39.7)	
Bystander CPR	842/1926	(43.7)	2118/4671	(45.3)	0.23
Bystander AED	41/1857	(2.2)	117/4499	(2.6)	0.36
**Initial rhythm confirmed by EMS**					0.13
VF	150/1912	(7.8)	338/4587	(7.4)	
Pulseless VT	10/1912	(0.5)	12/4587	(0.3)	
PEA	466/1912	(24.4)	1056/4587	(23.0)	
Asystole	1146/1912	(59.9)	2881/4587	(62.8)	
Others	140/1912	(7.3)	300/4587	(6.5)	
**Rhythm change during transportation**					
to VF	120/1757	(6.8)	321/4390	(7.3)	0.51
to pulseless VT	23/1730	(1.3)	42/4361	(1.0)	0.21
to PEA	350/1776	(19.7)	909/4406	(20.6)	0.41
to asystole	450/1815	(24.8)	1126/4442	(25.3)	0.65
to others	139/1737	(8.0)	287/4317	(6.6)	0.06
**Prehospital treatment by EMS**					
Defibrillation	234/1977	(11.8)	562/4673	(12.0)	0.83
Intravenous access	745/1957	(38.1)	1920/4279	(40.6)	0.05
Epinephrine	581/1948	(29.8)	1508/4717	(32.0)	0.09
Bag valve mask	317/1731	(18.3)	707/3922	(18.7)	0.80
**Advanced airway management by EMS**					< 0.01
Supraglottic airway device	898/1858	(25.8)	2095/4537	(46.2)	
Tracheal intubation	114/1858	(6.1)	425/4537	(9.4)	
Mechanical chest compression	186/2015	(9.2)	574/5023	(11.4)	0.01
EMS response time, minutes	7.0	(5.0–9.0)	8.0	(6.0–10.0)	< 0.11
EMS scene time, minutes	14.0	(10.0–19.0)	15.0	(11.0–19.0)	0.21
EMS transport time, minutes	10.0	(7.0–15.0)	11.0	(7.0–16.0)	< 0.01
EMS call-to-hospital time, minutes	33.0	(27.0–40.0)	35.0	(29.0–43.0)	< 0.01
**Rapid response car/helicopter**	185/2015	(9.2)	319/5023	(6.4)	< 0.01
Epinephrine	151/152	(99.3)	261/263	(99.2)	0.91
Amiodarone	10/152	(6.6)	22/263	(8.4)	0.51
Tracheal intubation	122/172	(70.9)	240/305	(78.7)	0.05

Data are shown as the number of positive observations/total number of observations (%) or as median (interquartile range). For each variable, the number of missing observations can be obtained as the difference between the total number of patients in each group and the total number of observations

EMS, emergency medical services; CPR, cardiopulmonary resuscitation; AED, automated external defibrillation; VF, ventricular fibrillation; VT, ventricular tachycardia; PEA, pulseless electrical activity

### In-hospital information and treatments

The patient’s status at hospital arrival and treatments performed in the hospital are given in Table 3. The proportions of ventricular fibrillation on hospital arrival were lower (3.2% vs. 4.4%) and the proportions of ROSC on hospital arrival were lower (9.2% vs. 12.0%) in the post- versus pre-pandemic group. We found no statistical differences in use of defibrillation (9.7% vs. 10.3%; *p* = 0.43), extracorporeal membrane oxygenation (ECMO) (3.4% vs. 4.2%; *p* = 0.14), and temperature control therapy (5.7% vs. 6.5%; *p* = 0.16). However, the receipt of emergency coronary angiography (5.0% vs. 7.2%; *p* < 0.01) and epinephrine were lower (71.3% vs. 74.6%; *p* < 0.01) in the post- versus pre-pandemic group.

**Table 3:** Demographics on hospital arrival and in-hospital treatments of patients before and after the COVID-19 pandemic

Variable	Pre-pandemic (*n* = 2015)		Post-pandemic (*n* = 5023)		p-value
**ECG on hospital arrival**					< 0.01
VF	89/2015	(4.4)	163/5023	(3.2)	
Pulseless VT	7/2015	(0.3)	17/5023	(0.3)	
PEA	388/2015	(19.3)	912/5023	(18.2)	
Asystole	1290/2015	(64.0)	3461/5023	(68.9)	
ROSC	241/2015	(12.0)	463/5023	(9.2)	
BT on hospital arrival, ºC	35.6	(34.6–36.3)	35.8	(34.8–36.4)	0.05
**GCS on hospital arrival**					0.31
3	1951/2015	(96.8)	4886/5023	(97.3)	
4–15	64/2015	(3.2)	137/5023	(2.7)	
**Blood tests**					
Lactate (mmol) †	1.8	(1.3–143.0)	1.9	(1.3–150.0)	0.11
WBC (10^3^/µL)	9.8	(7.3–12.7)	9.4	(7.0-12.5)	< 0.01
Hb (g/dL)	12.0	(10.0-13.7)	11.9	(10.0-13.8)	0.35
pH	6.85	(6.72–6.99)	6.85	(6.72–6.98)	0.66
PaCO_2_ (mmHg)	86.4	(66.4–113.0)	87.8	(66.3–114.0)	0.19
PaO_2_ (mmHg)	33.1	(18.0-68.3)	33.0	(18.2–64.0)	0.48
HCO_3_ (mEq/L)	15.1	(11.1–18.9)	15.3	(11.6–18.9)	0.05
BE (mEq/L)	-19.0	(-24.0 to -13.3)	-19.1	(-24.4 to -13.8)	0.05
Mechanical chest compression	311/2015	(15.4)	833/5023	(16.6)	0.34
Defibrillation	207/2008	(10.3)	484/4997	(9.7)	0.43
ECMO	84/2015	(4.2)	173/5023	(3.4)	0.14
Coronary angiography	146/2015	(7.2)	252/5023	(5.0)	< 0.01
Coronary intervention	62/140	(44.3)	141/250	(56.4)	0.02
IABP	52/2015	(2.6)	95/5023	(1.9)	0.07
Temperature control	127/1943	(6.5)	282/4985	(5.7)	0.16
**Medications**					
Epinephrine	1503/2015	(74.6)	3581/5023	(71.3)	< 0.01
Amiodarone	121/2015	(6.0)	238/5023	(4.7)	0.03
Atropine	23/2015	(1.1)	25/5023	(0.5)	< 0.01
Magnesium sulfate	19/2015	(0.9)	25/5023	(0.5)	0.03
Nifekalant	9/2015	(0.4)	22/5023	(0.4)	0.96
Lidocaine	9/2015	(0.4)	17/5023	(0.3)	0.50
Vasopressin	6/2015	(0.3)	1/5023	(0.0)	< 0.01

Data are shown as the number of positive observations/total number of observations (%) or as median (interquartile range). For each variable, the number of missing observations can be obtained as the difference between the total number of patients in each group and the total number of observations

ECG, electrocardiogram; VF, ventricular fibrillation; VT, ventricular tachycardia; PEA, pulseless electrical activity; ROSC, return of spontaneous circulation; BT, body temperature; GCS, Glasgow coma scale; WBC, white blood cells; Hb, hemoglobin; pH, potential hydrogen; pCO_2_, carbon dioxide partial pressure; pO_2_, oxygen partial pressure; HCO_3_, bicarbonate ion; BE, base excess; ECMO, extracorporeal membrane oxygenation; IABP, intra-aortic balloon pumping

†Lactate was measured in 1349 patients in the pre-pandemic group and 3371 patients in the post-pandemic group

### Outcomes

As can be seen in Table 4, the proportions of ROSC during transportation in the post-pandemic group were lower than those in the pre-pandemic group (8.9% vs. 11.5%, *p* < 0.01), and the mortality in the emergency department in the post-pandemic group was higher (81.9% vs. 74.8%, *p* < 0.01) than those in the pre-pandemic group. Mortality at 1 month in the post-pandemic group was higher than those in the pre-pandemic group (7.2% vs. 5.6%, *p* = 0.01) and the proportions of favorable outcome at 1 month in the post-pandemic group was lower (2.7% vs. 3.9%, *p* < 0.01, respectively) than those in the pre-pandemic group.

**Table 4:** Outcomes of patients before and after the COVID-19 pandemic

Variable	Pre-pandemic (*n* = 2015)		Post-pandemic (*n* = 5023)		p-value
**ROSC**					< 0.01
Prehospital ROSC	232/2015	(11.5)	448/5023	(8.9)	
ROSC after hospital arrival	438/2015	(21.7)	915/5023	(18.2)	
**Patient status**					< 0.01
Hospital admission	507/2015	(25.2)	910/5023	(18.1)	
Died in ED	1508/2015	(74.8)	4113/5023	(81.9)	
Survival at 1 month	143/1995	(7.2)	279/4982	(5.6)	0.01
Favorable outcome (CPC 1, 2) at 1 month	77/1953	(3.9)	134/4933	(2.7)	< 0.01
Favorable outcome at 3 months	66/1932	(3.4)	128/4908	(2.6)	0.07

Data are shown as the number of positive observations/total number of observations (%). For each variable, the number of missing observations can be obtained as the difference between the total number of patients in each group and the total number of observations

ROSC, return of spontaneous circulation; ED, emergency department; CPC, cerebral performance category

### Logistic regression analysis

Table 5 shows that the presence of a witness (witness by EMS: odds ratio [OR], 8.72; 95% confidence interval [CI] 5.49 to 13.86; *p* < 0.01; witness by citizen: OR 3.43; 95% CI 2.62 to 4.48; *p* < 0.01), bystander CPR (OR 1.99; 95% CI 1.48 to 2.67; *p* < 0.01), and shockable rhythm (OR 5.03; 95% CI 3.58 to 7.07; *p* < 0.01) were associated with survival of patients with OHCA. Increased age (OR 0.98; 95% CI 0.98 to 0.99; *p* < 0.01) and prolongation of EMS time from call to hospital (OR 0.98; 95% CI 0.97 to 1.00; *p* < 0.01) were associated with increased mortality. The timing when the OHCA happened, that is post-COVID-19 pandemic or pre-COVID-19 pandemic, was not associated with improved survival after adjusting for these factors (OR 0.86; 95% CI 0.67 to 1.09; *p* = 0.21).

**Table 5:** Multiple logistic regression analysis of survival at 1 month risk among out-of-hospital cardiac arrest patients after adjusting for within-hospital clustering

Variable	Original data			After multiple imputation		
	OR	95% CI	p-value	OR	95% CI	p-value
Post-COVID-19 pandemic	1.04	0.81–1.32	0.77	0.86	0.67–1.09	0.21
Pre-COVID-19 pandemic	1			1		
Age	0.98	0.98–0.99	< 0.01	0.98	0.98–0.99	< 0.01
Male	0.88	0.72–1.08	0.24	0.90	0.74–1.10	0.31
Female	1			1		
Other place	0.84	0.43–1.64	0.62	0.83	0.48–1.47	0.52
Nursing home	0.79	0.49–1.29	0.36	0.71	0.33–1.15	0.16
Public	1.60	1.20–2.13	< 0.01	1.47	1.09–1.98	0.01
Home	1			1		
Witness: EMS	8.34	5.28–13.33	< 0.01	8.72	5.49–13.86	< 0.01
Witness: citizen	3.42	2.58–4.53	< 0.01	3.43	2.62–4.48	< 0.01
Witness: none	1			1		
Bystander CPR +	2.04	1.51–2.76	< 0.01	1.99	1.48–2.67	< 0.01
Bystander CPR–	1			1		
EMS time from call to hospital	0.98	0.97–0.99	0.01	0.98	0.97-1.00	< 0.01
Shockable rhythm	1.64	1.12–2.41	< 0.01	5.03	3.58–7.07	< 0.01
Non-shockable rhythm	1			1		

OR, odds ratio; CI, confidence interval; EMS, emergency medical services; CPR, cardiopulmonary resuscitation

## Discussion

In the current analysis from a multicenter prospective cohort study, mortality and proportions of favorable outcome at 1 month declined among OHCA patients, EMS call-to-hospital time was longer, and the proportion of advanced airway management increased in the post-pandemic period relative to the pre-pandemic period; however, most prehospital and in-hospital treatments did not differ between the pre- and post-pandemic groups. Additionally, age, presence of a witness, bystander CPR, shockable rhythm, and EMS rescue time could predict the prognosis of OHCA.

The strength in this study is the study design; this study was a multicenter survey that contained precise information regarding patient characteristics and demographics, prehospital EMS treatments and physician-provided care, intensive care at the hospital, and mortality and neurological outcomes. Almost all previous studies in this area of interest have reported results focused on prehospital information or results from small group studies [3–6]. In contrast, the current study included data on all components in the chain of survival for OHCA with a large sample size; hence, our results more precisely reflected the effect of the COVID-19 pandemic on OHCA management.

We showed that duration of EMS rescue has become longer in the post-pandemic time compared with before the COVID-19 pandemic, which may have been related to the increasing mortality. The same trend has been reported in other countries [3, 4, 15–17]. Previous studies reported that the burden of infection during the COVID-19 pandemic affected EMS systems and delayed arrival and response times for all cases, including OHCA [18, 19]. Another study reported that longer EMS response times were associated with poorer outcomes in OHCA patients [14]; therefore, the EMS system should be improved to maintain response time within an average range in a disaster situation such as the COVID-19 pandemic.

The logistic regression analysis demonstrated some risk factors for survival among OHCA patients: presence of a witness, bystander CPR, shockable rhythm, age, and EMS time, and the results, even in the COVID-19 era, were similar to previous studies [4, 5, 14]. Of these five factors, bystander CPR and EMS time were factors that could be improved. Early in the pandemic, the healthcare system was coping not only with patients COVID-19 but also with the wider effects of the pandemic on the healthcare system in general like in times of disaster; however, the most difficult time has passed and now we have been trying to coexist with COVID-19. It is important to learn from this pandemic to prepare for future pandemics or major disasters to prevent further healthcare and medical insufficiencies. We have already discussed EMS rescue duration, but deepening our understanding of the chain of survival, improving public education on basic life support methods, and establishing sustainable medical systems are important social issues, especially in preparing for disaster situations.

Previous studies have aimed to evaluate proportions of prehospital ROSC as a main outcome, and the proportions declined in the post-pandemic group in this study, similar to previous studies [3, 4, 15, 16]. As for prehospital factors, our study demonstrated that baseline activities of daily living (ADL) were worsened, EMS call-to-hospital time was prolonged, and advanced airway management performed by paramedics increased in the post-pandemic group versus the pre-pandemic group. However, OHCA witnessed by others, bystander CPR, initial rhythm, and other interventions by EMS providers did not differ between groups, and most OHCA occurred at home. Lim et al. provided some explanations as follows: a lower proportion of bystander interventions occurred, EMS workflows changed during the COVID-19 pandemic, and patients may have been sicker, particularly in terms of heart disease, during the pandemic [16]. Although the quarantine in Japan was mild, frail people may have become weaker because of social activity restrictions. Therefore, preventing disease, frailty, and sarcopenia could be significant factors in improving OHCA prognosis. However, we did not adjust for these factors and could not assess the relationship between baseline patient health status and proportions of prehospital ROSC.

### Limitations

This study has some limitations. First, the target of the SOS-KANTO database was OHCA patients who were transported to the hospital; therefore, we did not assess all OHCA cases that occurred in this region. Second, we analyzed data collected through March 2021; however, the results may have been different if we evaluated data after April 2021, considering the spread of COVID-19, the overload on medical systems, and the worsened medical insufficiencies. Third, these results may have not be generalizable to other regions or countries where the medical and social systems are different from those in Kanto, Japan.

## Conclusions

The results of the current study showed that, in the post-pandemic group, mortality and favorable outcome rate 1 month after ROSC following OHCA worsened, EMS response time was prolonged, and advance airway management by EMS increased; however, most prehospital and in-hospital management did not change between pre- and post-pandemic. Our findings suggested that age, presence of a witness, bystander CPR, initial shockable rhythm, and EMS rescue time are prognostic factors for OHCA from the SOS-KANTO 2017 study.

### Electronic supplementary material

Below is the link to the electronic supplementary material.

**Supplementary Material 1:** List of members of the SOS-KANTO 2017 Steering Committee

## Data Availability

The data used in this study are available from the SOS-KANTO 2017 study group. However, these data were restricted for use under the license for the current study, and are not publicly available. Data are available from the authors upon reasonable request and with the permission of the SOS-KANTO 2017 study group.

## Abbreviations

OHCA: out-of-hospital cardiac arrest
the COVID-19: the SARS-CoV-2
EMS: emergency medical service
CPR: cardiopulmonary resuscitation
AED: automated external defibrillator
CA: cardiac arrest
the CPC: the cerebral performance category
ROSC: return of spontaneous circulation
IQR: interquartile range
CI: confidence interval
ADL: activities of daily living
