# Supplementary material for: Impact of the COVID-19 pandemic on prehospital and in-hospital treatment and outcomes of patients after out-of-hospital cardiac arrest: a Japanese multicenter cohort study
Source: BMC Emerg Med. 2024 Jan 8;24:12. doi: 10.1186/s12873-024-00929-8 (PMC10775511; doi:10.1186/s12873-024-00929-8)
Supplement: Supplementary file 1 — Supplementary Material 1: List of members of the SOS-KANTO 2017 Steering Committee [file 12873_2024_929_MOESM1_ESM.docx]

**Members of the SOS-KANTO 2017 Steering Council**

Munekazu Takeda: Tokyo Women's Medical University

Nobuya Kitamura: Kimitsu Chuo Hospital

Taka-aki Nakada: Chiba University Hospital

Hideo Yasunaga: The University of Tokyo

Shotaro Aso: The University of Tokyo

Takashi Tagami: Nippon Medical School Musashikosugi Hospital

Yosuke Honma: Chiba Kaihin Municipal Hospital

Yosihisa Tateishi: Chiba Kaihin Municipal Hospital

Tomoko Ogasawara: Nippon Medical School Hospital

Kei Hayashida: Keio University Hospital

Hiraku Funakoshi: Tokyo Bay Urayasu/Ichikawa Medical Center

Tomohisa Nomura: Juntendo University Nerima Hospital

Masaru Suzuki: Tokyo Dental College Ichikawa General Hospital

Kazuhiro Sugiyama: Tokyo Metropolitan Bokutoh Hospital

Atsushi Sakurai: Nihon University Hosapital
